# Exploiting high-throughput cell line drug screening studies to identify candidate therapeutic agents in head and neck cancer

**DOI:** 10.1186/2050-6511-15-66

**Published:** 2014-11-27

**Authors:** Anthony C Nichols, Morgan Black, John Yoo, Nicole Pinto, Andrew Fernandes, Benjamin Haibe-Kains, Paul C Boutros, John W Barrett

**Affiliations:** Department of Otolaryngology Head & Neck Surgery, Western University, London, Ontario Canada; Departments of Biochemistry and Applied Mathematics, Western University, London, Ontario Canada; Bioinformatics and Computational Genomics Laboratory, Institut de recherches cliniques de Montréal, Université de Montréal, Montreal, Quebec Canada; Department of Medical Biophysics, University of Toronto, Toronto, Canada; Informatics & Biocomputing Platform, Ontario Institute for Cancer Research, Toronto, Canada; Department of Pharmacology & Toxicology, University of Toronto, Toronto, Canada

**Keywords:** High throughput drug screening, Cell lines, Genomics, HNSCC, Mutations

## Abstract

**Background:**

There is an urgent need for better therapeutics in head and neck squamous cell cancer (HNSCC) to improve survival and decrease treatment morbidity. Recent advances in high-throughput drug screening techniques and next-generation sequencing have identified new therapeutic targets in other cancer types, but an HNSCC-specific study has not yet been carried out. We have exploited data from two large-scale cell line projects to clearly describe the mutational and copy number status of HNSCC cell lines and identify candidate drugs with elevated efficacy in HNSCC.

**Methods:**

The genetic landscape of 42 HNSCC cell lines including mutational and copy number data from studies by Garnett *et al*., and Barretina *et al*., were analyzed. Data from Garnett *et al*. was interrogated for relationships between HNSCC cells versus the entire cell line pool using one- and two-way analyses of variance (ANOVAs). As only seven HNSCC cell lines were tested with drugs by Barretina *et al*., a similar analysis was not carried out.

**Results:**

Recurrent mutations in human papillomavirus (HPV)-negative patient tumors were confirmed in HNSCC cell lines, however additional, recurrent, cell line-specific mutations were identified. Four drugs, Bosutinib, Docetaxel, BIBW2992, and Gefitinib, were found via multiple-test corrected ANOVA to have lower IC_50_ values, suggesting higher drug sensitivity, in HNSCC lines versus non-HNSCC lines. Furthermore, the PI3K inhibitor AZD6482 demonstrated significantly higher activity (as measured by the IC_50_) in HNSCC cell lines harbouring PIK3CA mutations versus those that did not.

**Conclusion:**

HNSCC-specific reanalysis of large-scale drug screening studies has identified candidate drugs that may be of therapeutic benefit and provided insights into strategies to target PIK3CA mutant tumors. PIK3CA mutations may represent a predictive biomarker for response to PI3K inhibitors. A large-scale study focused on HNSCC cell lines and including HPV-positive lines is necessary and has the potential to accelerate the development of improved therapeutics for patients suffering with head and neck cancer. This strategy can potentially be used as a template for drug discovery in any cancer type.

**Electronic supplementary material:**

The online version of this article (doi:10.1186/2050-6511-15-66) contains supplementary material, which is available to authorized users.

## Background

Despite advances in multi-modal treatment of head and neck squamous cell carcinoma (HNSCC), mortality rates for advance disease remain high [1]. Thus there is an urgent need to identify novel chemicals with high activity in this disease. As with other tumor types, however, the time- and resource-intensive, multi-step clinical trial process remains a tremendous barrier to rapid drug development. Moreover, only specific molecular subtypes of tumors may respond to any given target agent [2], thereby decreasing the number of patients eligible for a particular study.

Targeted therapy has become an important method in personalizing treatment for cancer patients based on the genetic mutations present in their tumor(s). Such therapies enable the use of drugs to specifically target molecules within the tumor that are responsible for the malignancy. A search of the literature, as well as clinical trials that are currently underway in HNSCC, revealed a variety of agents being investigated that target various cellular molecules (e.g. epidermal growth factor receptor [EGFR], members of the phosphatidylinositide 3-kinase [PI3K] pathway, mammalian target of rapamycin [mTOR], cyclin-dependent kinases, vascular endothelial growth factor receptor [VEGFR], retinoblastoma protein [pRB], toll-like receptors and Aurora kinases) (clinicaltrials.gov). However, despite the multiple trials, only EGFR tyrosine kinase inhibitors and EGFR monoclonal antibodies (e.g. cetuximab) have been approved for clinical use and demonstrate only modest activity in a subset of patients [3]. New strategies are needed not only to identify active molecules, but also to define the target population that is most likely to benefit from therapy.

Cell lines are imperfect models of cancer: they tend to be generated from more aggressive, often metastatic tumors, can demonstrate genetic and epigenetic changes relative to the parent tumors, and lack interactions with the surrounding stroma and immune system [4–7]. However, they remain an invaluable discovery tool as they provide an unlimited source of self-replicating material, are easily manipulated and can be screened in a cheap and high-throughput way with large panels of drugs. Moreover, relationships between drug sensitivity and tumor genotypes observed in patient samples are also reflected in cell lines [8].

The advent of next generation sequencing has allowed complete, affordable and rapid genomic characterization of both patient samples and of cell lines. In parallel, the development of high-throughput robotic drug screening platforms has facilitated the rapid testing of a large number of drugs. Together these techniques provide the ability to correlate mutation status, copy number variation and expression levels with drug response. Two recent, large-scale studies, involving hundreds of cell lines of different tissue types [8, 9] have confirmed well known genetic markers of drug response (e.g. response to *BRAF* inhibitors in *BRAF* mutant cell lines) and identified novel associations such as the marked sensitivity of Ewing’s sarcoma cells harboring the *EWS*-*FLI1* gene translocation to poly(ADP-ribose) polymerase (PARP) inhibitors [8]. However, given the large volume of data generated, only a limited analysis of the HNSCC cell lines involved in either study was presented. We endeavoured to reanalyze the data presented in these studies to provide a mutational landscape of HNSCC cell lines and to identify markers of drug sensitivity and resistance in HNSCC.

## Methods

### Defining the mutational and copy number landscape of HNSCC cell lines

**Figure 1:**
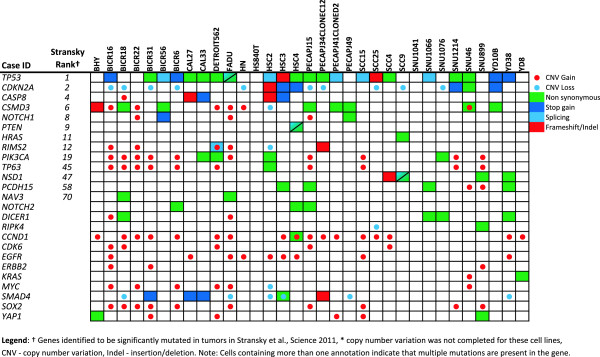
**Genetic landscape of head and neck cancer cell lines based on data from Barretina** ***et al***., **Nature 2012.**

**Figure 2:**
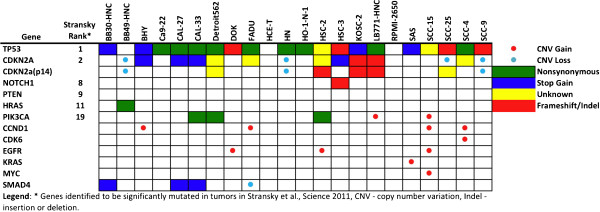
**Genetic landscape of head and neck cancer cell lines based on data from Garnett** ***et al***., **Nature 2012.**

The study by the Broad-Novartis group (Barretina *et al*.) included 31 HNSCC cell lines (of 947 total), seven of which were screened with 24 anticancer agents [9]. The cell lines were characterized by sequencing of ~1500 genes, as well as with array-based copy number variation (CNV) analysis and using mRNA abundance microarrays. A second study, by Garnett and coworkers, evaluated 639 cell lines (22 HNSCC lines) treated with 131 agents and characterized by targeted sequencing of 60 cancer genes, as well as array-based assessment of CNVs and mRNA abundance [8]. Note that eleven identically named HNSCC cell lines were common to both studies yielding a total of 42 uniquely named cell lines when both studies were combined. We integrated the CNV and mutational analysis of the most commonly altered genes from the two studies into Figures 1 and 2 and correlated them with the changes reported from patient samples by Stransky *et al*. [10]. CNV levels from Garnett *et al*., were simply reported as 0 (deletion), between 0 and 8 (copy-number neutral), and greater than 8 (amplification). Barretina *et al*. reported CNVs as continuous variables, relative to control genes with 0 considered “non-amplified”. We considered values greater than 2 (reflecting at least 2 extra gene copies) as amplifications and less than -2 (representing homozygous deletion) as this appeared to agree with the TCGA data from http://cbioportal.org and correspond best with the amplifications and deletions noted in the study by Garnett *et al*. (Additional file 1: Table S1).

### Identification of biomarkers of chemotherapeutic sensitivity and resistance in HNSCC cell lines

Due to the small number of HNSCC cell lines that were treated with drugs in Barretina *et al*. (7 lines), we restricted drug sensitivity analysis to the data from Garnett *et al*. [8]. All statistical analysis was performed with the R statistical environment, version 2.15.2 (R Foundation for Statistical Computing) with the fdrtool package [11] version 1.2.10, to control the rate of false discovery due to multiple testing. We compared the half-maximal inhibitory concentration (IC_50_ in μM) for each drug between HNSCC cell lines and non-HNSCC cell lines via one-way analysis of variance (ANOVA) as computed through *t*-tests. Specifically, for each drug *i*, cell lines were partitioned into two groups *j* = {HNSCC, non-HNSCC} as per their cell-line type. Letting *k* denote replicate number, the linear model for each *t*-test was the standard *y*_*ijk*_ = *μ*_*j*_ + *ε*_*ijk*_, where *y*_*ijk*_ represents the observed log_2_(IC_50_), *μ*_*j*_ represents the mean response of group *j*, and each *ε*_*ijk*_ represents a realization of . To control the false discovery rate, the “local false discovery rate” (LFDR) was estimated via computed *p*-values using Strimmer’s fdrtool [11, 12]. The LFDR has been championed by Efron and others for genomic studies as it is directly interpretable as posterior probability, and not a “corrected *p*-value” [13, 14]. A LFDR <0.05 was considered significant and a LFDR <0.1 was considered to be approaching statistical significance. We then looked for associations of copy number changes and mutations with response to drug treatment by two-way ANOVA including factor interaction, again using the LFDR to control false discovery rates. Specifically, the linear models used for the ANOVAs was *y*_*ijk*_ = *μ* + *α*_*i*_ + *β*_*j*_ + *γ*_*ij*_ + *ε*_*ijk*_ where group *i* = {copy-number unchanged, copy-number changed}, group *j* = {wild-type, mutant}, and *k* again denotes replicate number. As per standard ANOVA, *α*_*i*_ and *β*_*j*_ represent the mean additive responses of their respective groups, *γ*_*ij*_ represents any non-additive interaction effect, *ε*_*ijk*_ represents a realization of *ε* ~ *N*(0, *σ*^2^), and *μ* represents the grand-mean effect. The standard constraints ∑_*i*_*α*_*i*_ = 0, ∑_*j*_*β*_*j*_ = 0, and ∑_*ij*_*γ*_*ij*_ = 0 were used to ensure that all parameters of each model were identifiable.

## Results

### The genetic landscape of HNSCC cell lines is similar to HPV-negative tumors

The mutational landscape of the 42 HNSCC cell lines, all of which were HPV-negative [7], demonstrated similarities with primary tumor samples from HPV-negative patients; including frequent mutations in tumor suppressor genes *TP53* (74% of cell-lines [9]; 62% of tumors [10]) and *CDKN2A*, and less frequent ones in *PTEN*, *SMAD4*, *NOTCH1* and *NOTCH2* (Figures 1 and 2). Other similarities were rare activating mutations in oncogenes *PIK3CA* and *HRAS*, deletions of *CDKN2A* and amplifications of *CCND1*, epidermal growth factor receptor (*EGFR*), *MYC* and *PIK3CA*. A complete listing of mutations identified in HNSCC cell lines in Barretina *et al*. is provided in Additional file 2: Table S2. However, there were multiple, recurrent mutations in genes rarely or not identified in the patient samples (Additional file 2: Table S2 and Additional file 3: Table S3). In fact, there were 22 genes more frequently mutated than *TP53*, which was the most commonly mutated gene found in tumor samples (Additional file 3: Table S3). Most of these mutations were identical in all cell lines, such as two 5′ UTR mutations observed in neural cell adhesion molecule 1 (*NCAM1*) (insertion of adenine at position 112832307 (dbSNP ID: rs117108942) and deletion of cytosine at position 112832340) in virtually every HNSCC cell line in Barretina *et al*. Of note, 11 cell lines with the same name were characterized in both studies. However, two of these lines had significant discrepancies in terms of mutations between the studies (BHY, SCC9) bringing the true identities of the lines into question (Additional file 1: Table S1). Personal correspondence with the authors of Barretina *et al*. and the methods section of Garnett *et al*., have confirmed that the identification of their cell lines were confirmed with genotyping. The genotyping results are not provided in the supplementary data to allow direct comparison.

### Chemicals with high and low activity in HNSCC cell lines

**Figure 3:**
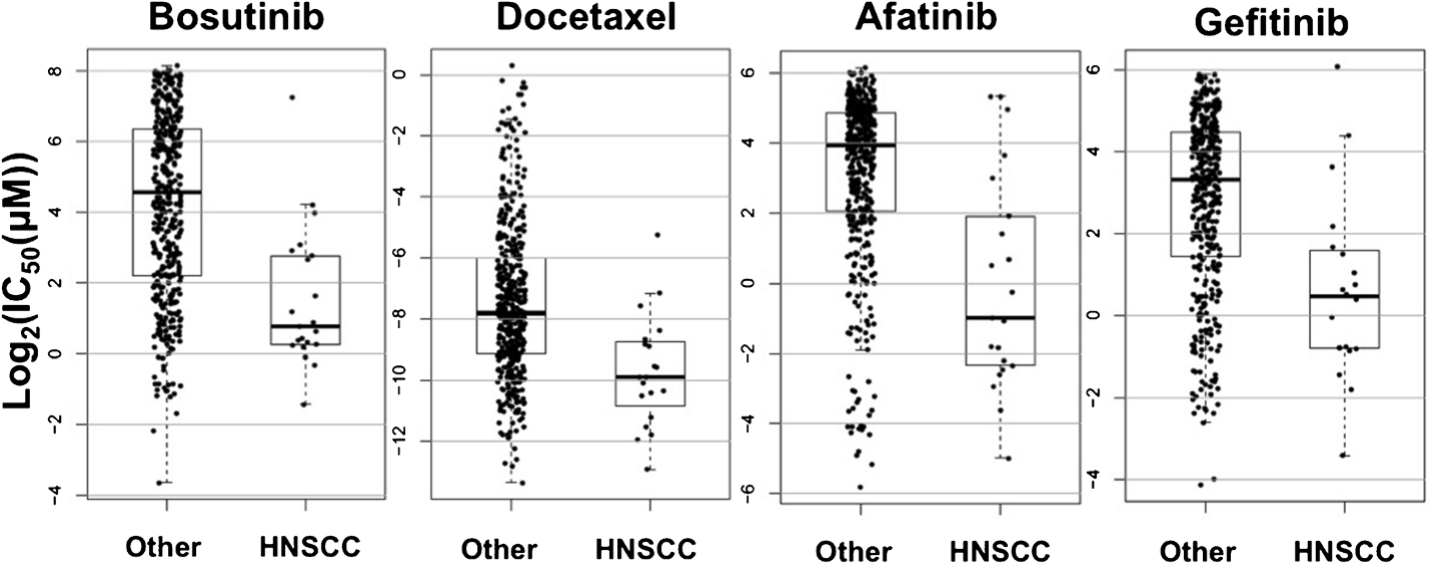
**Drug activity of HNSCC** ***vs.*** **non-** **HNSCC** **(“Other”)** **cell lines from Garnett** ***et al*** **.,** **Nature 2012.** Points represent individual observations, while boxes show estimates of the respective median, interquartile range, and extrema.

Four chemicals, Docetaxel (anti-mitotic chemotherapy), Bosutinib (combined SRC/ABL inhibitor), Afatinib (an EGFR and HER2 inhibitor), and Gefitinib (an EGFR inhibitor) were found to have significantly increased activity in HNSCC cell lines compared with the remainder of the cell line pool (Table 1, Figure 3). Two drugs, methotrexate and PD-173074 (an inhibitor of the fibroblast growth factor receptor [FGFR] and VEGFR) were found to have significantly lower activity in HNSCC lines (Table 1). A complete listing of the associations between drug response and HNSCC cell line type can be found in Additional file 4: Table S4.

**Table 1:** **Drugs demonstrating significantly increased or decreased activity in HNSCC cell lines compared with non**-**HNSCC lines**

Drug	p*	LFDR**	Drug response relative to other cell lines	Effect(Log2(IC50(μM))	95% CI (Log2(IC50(μM)))
Bosutinib	<0.0001	0.0015	**Sensitive**	-2.74	(-3.67 to -1.82)
Docetaxel	<0.0001	0.0161	**Sensitive**	-2.29	(-3.17 to -1.42)
BIBW2992	0.0002	0.0161	**Sensitive**	-3.12	(-4.53 to -1.71)
Gefitinib	0.0003	0.0258	**Sensitive**	-2.18	(-3.24 to -1.12)
PD-173074	0.0002	0.0183	**Resistance**	0.95	(0.50-1.40)
Methotrexate	0.0005	0.0258	**Resistance**	1.59	(0.77-2.42)

Legend: * - calculated by one way analysis of variance (ANOVA), LFDR - local false discovery rate, ** - calculated by the Strimmer method, IC50 - half maximal inhibitory concentration, CI - confidence interval.

Of note, there were five identically named cell lines in both studies that were tested with identical drugs (Additional file 5: Table S5), four of which had similar mutational profiles suggesting that they were indeed identical lines. SCC-9 was excluded due to discrepancies in the mutational profiles reported by Barretina *et al*. and Garnett *et al*. (Additional file 1: Table S1). We sorted the comparable IC50s into three groups, representing cell lines that were exquisitely sensitive (IC50 < 3 μM) to the drug, responders (IC50 3.1-7.9 μM) and resistant cell lines (IC50 > 8 μM). We found that the majority were comparable (Additional file 5: Table S5).

### Activating PIK3CA mutations are correlated with response to the PI3K inhibitor, AZD6482

**Figure 4:**
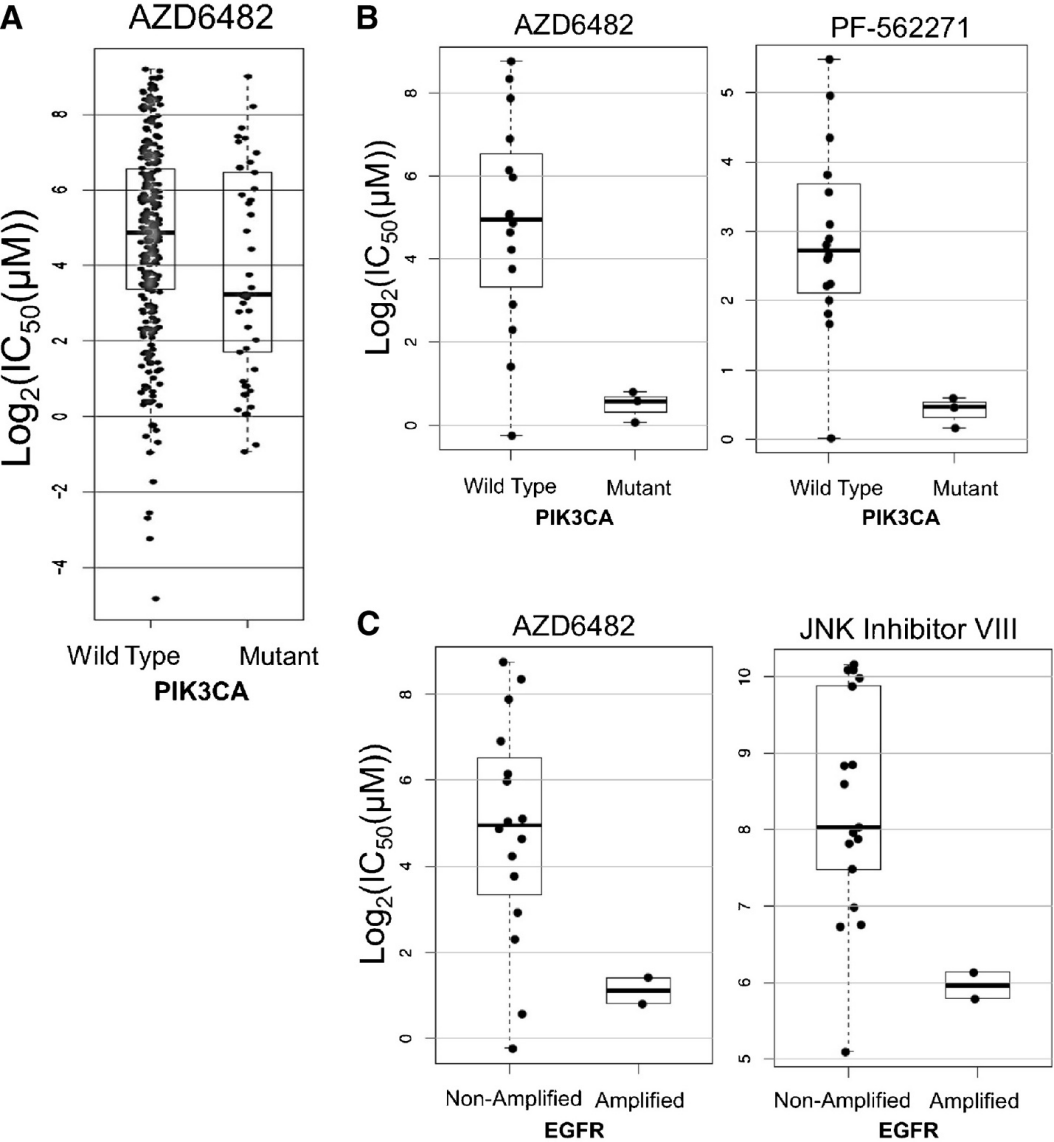
**Drugs with differential activity by mutational status. (A)** PI3K inhibitor AZD6482 demonstrates increased activity in PIK3CA mutant versus wild-type cell lines. **(B)** When analysis was restricted to HNSCC cell lines, AZD6482 and FAK inhibitor PF-562271 demonstrated increased activity in PIK3CA mutant lines. **(C)** AZD6482 and JNK Inhibitor VIII had increased activity in EGFR amplified cell lines relative to wild-type lines. Points represent individual observations, while boxes show estimates of the respective median, interquartile range, and extrema.

The complete listing of drug sensitivity and gene status can be found in Additional file 6: Table S6, with the significant findings summarized in Table 2. Only mutations and copy number changes in *EGFR*, *TP53*, *CDKN2A*, *PIK3CA* and *SMAD4* were present with sufficient frequency (>10%) in the cell lines to allow analysis. It should be noted that not all cell lines were treated with every drug and some genetic changes occurred in a very small number of cell lines, which resulted in exclusion of analysis of certain drugs with a particular genetic change. Of the 131 drugs tested, three were PI3K inhibitors including AZD6482, GDC0941, and the combined mTOR and PI3K inhibitor NVP-BEZ235. We calculated a robust increase in sensitivity to AZD6482, explainable by the interaction of *PIK3CA* mutation status and HNSCC cell-line type (*LFDR* <0.023, Figure 1). In addition, an increase in AZD6482 sensitivity was shared by all *PIK3CA* mutants (*p* <0.037, Figure 4A) regardless of cell line type. No association was observed for the other PI3K inhibitors, GDC0941 and NVP-BEZ235, and *PIK3CA* mutation status (LFDR ≈ 1). There were too few *PIK3CA* amplified cell lines to examine the effect of amplification alone on drug response, however when these were pooled with the *PIK3CA* mutant lines, no drugs were found to be preferentially active when compared to *PIK3CA* wild-type cell lines (Additional file 6: Table S6).

**Table 2:** **Significant associations of mutations and amplifications with drug response in HNSCC cell lines**

Drug	Gene	Comparison	p	LFDR	Mean WT/NA log_2_(IC50(μM))	Mut/Amp log_2_(IC50(μM))	Effect Log2(IC50(μM)) [95% CI]
ATRA	*TP53*	wild-type vs mutant	<0.001	0.0069	9.96	7.15	2.81 [1.82-3.79]
AZD6482	*PIK3CA*	wild-type vs mutant	<0.001	0.023	4.87	0.689	4.18 [2.73-5.63]
JNK Inhibitor VIII	*EGFR*	non-amplified *vs*. amplified	<0.001	0.056	8.31	5.96	2.34 [1.49-3.20]
AZD6482	*EGFR*	non-amplified *vs*. amplified	<0.001	0.056	4.83	1.11	3.72 [2.17-5.26]
PF-562271	*PIK3CA*	wild-type vs mutant	<0.001	0.079	2.88	0.379	2.50 [1.55-3.46]

Legend: p – p value testing interaction, *LFDR* - local false discovery rate, *WT* - wild-type, *NA* - non-amplified, Mut – mutation, Amp – amplified, *CI* - confidence interval.

When examining responses to inhibitors of upstream and downstream members of the *PIK3CA* pathway, the FAK inhibitor PF-562271 (upstream) demonstrated a trend towards selective inhibition of *PIK3CA* HNSCC mutant cell lines (LFDR = 0.079, Figure 4B), while no effect was observed for downstream inhibitors including three AKT inhibitors (AKT inhibitor VIII, MK-2206, A-443654) and four mTOR inhibitors (Rapamycin, Temsirolimus, JW-7-52-1, AZD8055).

There was a trend towards increased sensitivity to AZD6482 and JNK Inhibitor VIII in cell lines with EGFR amplifications (LFDR = 0.056, Figure 4C). The strongest association observed was increased activity of the retinoid receptor antagonist ATRA in *TP53* mutant lines (LFDR = 0.007, Table 2 and Additional file 6: Table S6).

## Discussion

Following decades of active research, only one class of targeted molecular agents, epidermal growth factor receptor (EGFR) inhibitors, have been approved for use in head and neck cancers [15]. Despite a modest survival benefit when administered concurrently with radiation, response rates to EGFR inhibitors are low when given alone (13%) and of limited duration (2–3 months). More effective drugs are needed in order to improve outcomes and reduce treatment-induced morbidities for HNSCC patients.

By pairing next-generation sequencing of cell lines with high-throughput drug screening techniques, the impressive studies by Garnett *et al*. and Barretina *et al*. [8, 9], confirmed, in multiple tissue types, known associations of genetic alterations with drug sensitivity and uncovered a multitude of new ones. Their sequencing findings were in agreement with preliminary data from The Cancer Genome Atlas (TCGA) HNSCC study, where a multitude of potentially druggable targets including amplifications (e.g. *FGFR1*, *CCND1*, *MYC*, *EGFR*), deletions (e.g. *PTEN*), activating mutations (e.g. *PIK3CA*) and fusions (e.g. *FGFR3*/*TACC3*) were observed. Despite a relatively limited number of cell lines, our HNSCC-specific reanalysis of these studies shows that the spectrum of mutations observed in HNSCC cell lines is similar to that of primary HNSCC patient samples [10, 16]. Overall, mutations in tumor suppressor genes, such as *TP53* are frequent, while activating mutations in oncogenes (*PIK3CA*, *HRAS*) occur at lower frequencies.

As mentioned, our analysis revealed 22 genes that are more frequently mutated than *TP53*, which is the most commonly mutated gene found in patient tumor samples (Additional file 3: Table S3). This discrepancy may be partially explained by the fact that mutations in introns and 5′ and 3′ untranslated regions (UTRs) were reported for the cell lines but not for the patient samples. In addition, there were multiple, identical mutations noted in cell lines, such the mutations observed in *NCAM1*[9]. Interestingly, *NCAM1* is known to signal through FAK, a direct target of PF-562271, which may contribute to its observed activity in *PIK3CA*-mutant HNSCC cell lines. However, we speculate that the near identical mutations found in these 22 highly mutated genes are germ-line variants and/or artifacts of cell line establishment. This inability to distinguish the cause of mutation(s) highlights the importance of comparing tumor sample or cell line DNA, to matched-normal tissue. Moving forward, it is crucial that matched-normal tissue and blood samples, representing the germ-line genetic profile are obtained when cell lines are established for improved sequencing analyses.

The most obvious directly druggable target in HNSCC appears to be the PI3K pathway, which was mutated in 8% of the patient samples examined by Stransky *et al*. [10]. We noted that activating *PIK3CA* mutations appeared to be more frequent in three HPV-positive tumors sequenced by Stransky *et al*., and we confirmed this finding by sequencing the *PIK3CA* hotspots in a larger number of HPV-positive (46) and negative (43) oropharyngeal HNSCC samples [17]. We found that *PIK3CA* mutations occurred in 28% of the HPV-positive oropharyngeal tumors versus 10% of the HPV-negative samples, confirming that this is an important therapeutic target in HNSCC. There is great interest in targeting PI3K as it is frequently either amplified or mutated in a large variety of human cancers [18], however the selection of the best possible drug is critical before moving forward with clinical studies. Selection of the appropriate agent is crucial because different drugs, which might target the same molecule, can display extremely variable potencies based on a variety of factors including a drug’s binding site, delivery efficiency, half-life and metabolic interactions. An additional limitation of our analysis in terms of drug development progressing to routine patient care is the low number of drugs tested against the cell lines. Increasing the number of drugs tested, including drugs targeting identical molecules, would help ensure that potent drugs are identified and cellular targets further verified in the most appropriate patient population.

Cell lines are not perfect models of cancer due to their lack of three-dimensional stromal environment, lack of interactions with an immune system, and an inability to test drug delivery issues. Many of these concerns can be overcome in xenografts and human testing. The utility of cell line drug screening, given that poor correlation of drug testing in matched cell lines and patients have variably been observed [19, 20] while patient derived xenografts appear to preserve nearly all the molecular feature of the original tumor. Emerging data suggests that while many cell lines may not recapitulate the molecular landscape of primary tumors, selecting cell line models with comparable genetic profiles will yield more accurate drug screening results [21–23]. With appropriate cell line models selected, cell line screening can be a robust, rapid and inexpensive preliminary screen before proceeding with more expensive and ethically-challenging animal and human studies. In our study, only molecules affecting the PI3K pathway (AZD6482 and PF-562271) were correlated with selective inhibition of *PIK3CA* mutant lines. However, it is somewhat surprising that AZD6482 was the most tightly correlated with *PIK3CA* mutation status as it has approximately an 87-fold higher affinity for PI3Kβ than PI3Kα [24]. It is noteworthy that AZD6482 appeared significantly more effective than the pan PI3K inhibitor GDC0941 (LFDR ≈ 1), which has a markedly higher affinity for PI3Kα [24, 25] indicating that binding affinity may not be perfectly correlated with *in vitro* activity, much less *in vivo* efficacy. As well, compounds such as NVP-BEZ235, a PI3K/mTOR inhibitor have been previously found to have selective activity in *PI3KCA*-mutant HNSCC cell lines and patient-derived xenografts (PDX) [26]. However, NVP-BEZ235 activity did not correlate to *PI3KCA* mutant cell lines in these studies, reinforcing the importance of identifying the most promising preclinical candidates that have the strongest correlation with genomic changes to take forward into clinical trials.

There are two highly specific PI3Kα inhibitors, GDC-0032 (Genetech) and BYL719 (Novartis) that have demonstrated superior inhibition of *PIK3CA* mutant and amplified cell lines as well as tumor xenografts [27, 28]. Indeed, BYL719 was screened against the CCLE cell line pool despite not being included in the report by Barretina *et al*. [28]. Given these promising preclinical studies, both compounds have been carried forward into phase I trials, and the study for BYL719 has been completed [27, 28]. The trial included patients with solid tumors harboring *PIK3CA* amplifications and/or mutations including 8 HNSCC patients, six of which had stable disease and two had partial responses (25%) [28]. A phase II study in recurrent/metastatic HNSCC is already underway (http://clinicaltrials.gov/ct2/show/NCT01602315). BYL719 is an excellent example of a molecule initially identified in studies using cell lines, its efficacy confirmed in PDX models and results of the first Phase I clinical trials are now in the literature [29, 30]. This process highlights the potential of this “bench to bedside” approach using high throughput platforms to identify effective anticancer agents. As targeted therapy for cancer treatment continues to develop, this bench to bedside approach will enable researchers to screen large numbers of drugs against a multitude of cancer cell lines with the goal of confirming successful agents in PDX models. Drugs that are found to be effective in both cell lines and in mice combined with NGS for biomarker prediction association, will better enable us to accurately treat patients.

EGFR inhibitors have been integrated into routine clinical care for HNSCC patients based on the landmark Bonner study [31]. EGFR is the only targeted therapy approved for the treatment of head and neck cancer. Perhaps it is not surprising that two of the four EGFR inhibitors that were tested demonstrated significantly higher activity in HNSCC lines versus the rest of the cell line pool (Table 1). However, only a subset of patients benefit from these agents [15]. To better illustrate this, Erlotinib was one of the EGFR inhibitors not found to have significantly increased activity in HNSCC cell lines compared with other cancer line types, further emphasizing the importance of accurate drug selection.

The mechanisms of sensitivity and resistance to EGFR inhibitors in HNSCC are poorly understood [15]. In lung cancer, EGFR response is tightly correlated with activating *EGFR* mutations, while resistance in colon cancer is mediated by downstream KRAS mutations [15]. These genetic changes essentially never occur in HNSCC [10], and EGFR inhibitor response has not been correlated with EGFR amplification or expression [15, 31]. Indeed, in our study we failed to identify a genetic correlate of EGFR inhibitor sensitivity. An expanded study including a larger number of molecularly characterized HNSCC cell lines could potentially address this important clinical issue.

While EGFR amplification was not correlated with EGFR inhibitor efficacy, it was associated with increased response to the PI3K inhibitor AZD6482 and JNK inhibitor VIII. EGFR signalling is mediated through several pathways including PI3K and JNK [32, 33]. As EGFR amplifications do occur in HNSCC [10], this potentially relevant association should be explored further.

Ultimately our analysis is limited by the number of cell lines, which restrict the number of lines with any given genetic aberration(s) that could be tested. Another limitation is that not all drugs were tested in all cell lines. However, despite these limitations as well as discrepancies in drug responses described by Papillon-Cavanagh and colleagues [34], when comparing identical cell lines that were screened against the same compounds in the two studies, we found the results to be in agreement for many of the cell lines (Additional file 5: Table S5). There were 12 HNSCC cell lines displaying sensitivity to a compound (IC50 below 8 μM) in either or both of the studies. Of these 12 cases, ten were found to be very similar between the two studies (IC50 values within 3 μM) (Additional file 5: Table S5). In cases where cell lines displayed resistance, it was not possible for an exact comparison to be performed since Barretina *et al*. only screened drugs to a maximum of 8 μM, so many resistant cell lines are merely listed as having an IC50 of 8 μM. In contrast, Garnett *et al*. screened cell lines up to 3 orders of magnitude higher (10 mM). However, if we compare cell lines based on their characterization of resistance (IC50 above 8 μM) in their respective study, we find that the majority of cell line IC50 values were concordant between the two studies (Additional file 5: Table S5).

An additional limitation of our analysis is that all of the cell lines utilized were HPV-negative to the best of our knowledge [7]. Given the slow epidemic of HPV-related oropharyngeal cancer, this is a glaring omission as novel therapies for this patient cohort are of great interest to the head and neck oncology community. However, there are only 9 reported HPV-positive cell lines in the literature, all of which were derived from either recurrent tumors or smokers [35, 36], and are thus less likely to recapitulate the treatment-sensitive HPV-positive tumors encountered in clinical practice. We suggest that the development of further HPV-positive cell lines and their incorporation into large-scale HNSCC cell line drug screening studies has the potential to identify novel effective agents and the mechanisms of drug sensitivity and resistance in HNSCC. Hopefully, this will lead to significant improvements in survival that has eluded us to date.

The disagreements noted in the cell line sensitivities and mutations between the two studies have significant implications for future work of this type. They are many factors that can explain these discrepancies, however the most likely is that the identically named lines are in fact different, despite the fact that genotyping was completed in both studies. Other possible sources of disagreements in the data are differences in screening techniques, different drug concentrations, and different statistical models to calculate IC50 values from the dose–response curves. Ideally, the genotyping data can be compared to determine the discrepancies and provide the definitive genotype for cell lines. We also suggest that a standard methodology of cell line drug screening needs to be developed to allow external validation of future findings.

## Conclusions

High throughput drug screening of molecularly characterized HNSCC cell lines has the potential to rapidly identify promising agents to improve therapies for patients suffering with head and neck cancer. An expanded HNSCC specific study including HPV-positive cell lines has the potential to identify effective agents, as well as mechanisms of resistance and sensitivity to molecular agents.

### Ethics approval

No ethics approval was required as this present work represents an investigation and analysis of publicly accessible data.

## Electronic supplementary material

Additional file 1: Table S1: Comparison of mutations noted in the studies by Garnett *et al*. and Barretina *et al*. Legend: CNV - relative copy number variation, Disagreements highlighted in bold. (XLSX 46 KB)

Additional file 2: Table S2: Complete list of mutations found in the HNSCC cell lines in Barretina *et al*., Nature 2012. Legend: Del - deletion, Ins - insertion, 5′ UTR - 5′ untranslated region, 3′ UTR - 3′ untranslated region, SNP - single nucleotide polymorphism. (XLSX 231 KB)

Additional file 3: Table S3: Comparison of mutations noted in head and neck squamous cell tumors and cell lines. Legend: *Significantly mutated genes found in patient tumor samples from Stransky *et al*., Science 2011, **Most frequently mutated genes identified in cell lines in Barretina *et al*., Nature 2012. (XLSX 68 KB)

Additional file 4: Table S4: Associations of drug response and HNSCC cell line type versus non-HNSCC cell lines. Legend: t - t statistic calculated by ANOVA, p - p value calculated by ANOVA, LFDR - local falsed discovery rate, Effect - differences in the Log2(IC50(μM)) for HNSCC versus non-HNSCC cell lines, 95% lower limit and 95% upper limit delineate confidence intervals for the effect size. (XLSX 63 KB)

Additional file 5: Table S5: Comparison of drug sensitivities in cell lines common to the studies by Garnett et al. and Barretina et al. (DOCX 16 KB)

Additional file 6: Table S6: Associations of mutations and copy number alterations with drug response in HNSCC cell lines. Legend: wt - wild type, nor - normal (non-amplified), mt - mutation, amp - amplified, del - deletion, t - t statistic calculated by ANOVA, p - p value calculated by ANOVA, Wild-type: Log2(IC_50_(μM)) for wild-type cell lines, Other: Log2(IC50(μM)) for mutated and/or amplified cell lines, Effect: differences in the Log2(IC50(μM)) for WT/nor and mut/amplified cell lines, 95% lower limit and 95% upper limit delineate confidence intervals for the effect size, LFDR: Local false discovery Rate. (XLSX 120 KB)

## Abbreviations

HNSCC: Head and neck squamous cell carcinoma
HPV: Human papillomavirus
EGFR: Epidermal growth factor receptor
LFDR: Local false discovery rate
IC50: Inhibitory concentration, 50%
CNV: Copy number variation
UTR: Untranslated region
ANOVA: Analysis of variance.
