# Supplementary material for: Exploiting high-throughput cell line drug screening studies to identify candidate therapeutic agents in head and neck cancer
Source: BMC Pharmacol Toxicol. 2014 Nov 27;15:66. doi: 10.1186/2050-6511-15-66 (PMC4258049; doi:10.1186/2050-6511-15-66)
Supplement: Supplementary file 5 — Additional file 5: Table S5: Comparison of drug sensitivities in cell lines common to the studies by Garnett et al. and Barretina et al. (DOCX 16 KB) [file 40360_2014_350_MOESM5_ESM.docx]

**Supplemental Table 5** Comparison of drug sensitivities in cell lines common to the studies by Garnett et al. and Barretina et al

|  |  | **IC50 (μM)** | |
| --- | --- | --- | --- |
| **Cell Line** | **Compound** | **Barretina*** | **Garnett**** |
| CAL 27 | 17-AAG | 0.167 | 0.191 |
| CAL 27 | AZD6244 | 0.438 | 2.945 |
| CAL 27 | PD-0325901 | 0.134 | 0.043 |
| CAL 27 | PLX4720 | 8.000 | 313.250 |
| CAL 27 | Nutlin-3a | 8.000 | 32.622 |
| Detroit 562 | 17-AAG | 0.239 | 0.042 |
| Detroit 562 | AZD6244 | 8.000 | 64.264 |
| Detroit 562 | Nilotinib | 8.000 | 67.088 |
| Detroit 562 | PD-0325901 | 8.000 | 3.935 |
| Detroit 562 | PLX4720 | 8.000 | 142.025 |
| Detroit 562 | Nutlin-3a | 8.000 | 820.571 |
| FaDu | 17-AAG | 0.019 | 0.002 |
| FaDu | AZD6244 | 8.000 | 7.546 |
| FaDu | Nilotinib | 5.613 | 160.292 |
| FaDu | PD-0325901 | 2.066 | 0.896 |
| FaDu | PLX4720 | 8.000 | 97.320 |
| FaDu | Nutlin-3a | 8.000 | 712.657 |
| SCC-25 | 17-AAG | 0.092 | 0.244 |
| SCC-25 | AZD6244 | 8.000 | 6.032 |
| SCC-25 | PD-0325901 | 3.134 | 0.222 |
| SCC-25 | PLX4720 | 8.000 | 837.985 |
| SCC-25 | Nutlin-3a | 8.000 | 627.034 |

Legend: IC50 - half maximal inhibitory concentration; * and ** - IC50 measurements from Barretina et al., Nature 2012 and Garnett et al., Nature 2012, respectively. Light grey represents cell lines that are exquisitely sensitive (IC50 < 3 μM), medium grey signifies responders (IC50 3.1-7.9 μM) and dark grey are considered resistant (IC50 > 8 μM) Note that drugs were only tested up to 8 μM by Barretina and colleagues.
